# Vocational Rehabilitation with or without Work Module for Patients with Chronic Musculoskeletal Pain and Sick Leave from Work: Longitudinal Impact on Work Participation

**DOI:** 10.1007/s10926-020-09893-z

**Published:** 2020-05-06

**Authors:** Timo T. Beemster, Coen A. M. van Bennekom, Judith M. van Velzen, Monique H. W. Frings-Dresen, Michiel F. Reneman

**Affiliations:** 1 University Medical Center Groningen, Department of Rehabilitation Medicine, University of Groningen, Haren, P.O. Box 30.002, 9750 RA Groningen, The Netherlands; 2 Department of Research and Development, Heliomare Rehabilitation Center, Wijk aan Zee, The Netherlands; 3 Amsterdam UMC, Coronel Institute of Occupational Health, Amsterdam Public Health Research Institute, University of Amsterdam, Amsterdam, The Netherlands

**Keywords:** Chronic pain, Observational study, Occupational therapy, Biopsychosocial, Multidisciplinary

## Abstract

*Purpose* To study the longitudinal relationship between interdisciplinary vocational rehabilitation (VR) with and without additional work module on work participation of patients with chronic musculoskeletal pain and sick leave from work. *Methods* Retrospective longitudinal data retrieved from care as usual in seven VR centers in the Netherlands was used. The VR program without work module consisted of multi-component healthcare (physical exercise, cognitive behavioral therapy, education, relaxation). The other program with additional work module (VR+) included case management and a workplace visit. Generalized estimating equations using binary logistic was applied. The dependent variable was work participation (achieved/not achieved) on discharge and 6-months follow-up. Independent variables were type of intervention, return to work expectation, sick leave duration, working status, job strain, and job dissatisfaction. *Results* Data from N = 470 patients were analyzed, of which 26% received VR and 74% VR+. Both programs increased work participation at 6-months follow-up (VR 86%, VR+ 87%). The crude model showed a significant longitudinal relationship between type of intervention and work participation in favor of VR+ (OR 1.8, p = 0.01). The final model showed a non-significant relationship on discharge (OR 1.3, p = 0.51) and a significant relationship on 6-months follow-up in favor of VR+ (OR 1.7, p = 0.04). RTW expectation was a significant confounder in the final model on discharge and 6-months follow up (OR 3.1, p = 0.00). *Conclusions* Both programs led to increased work participation. The addition of a work module to the VR program lead to a significant increase in odds of work participation at 6-months follow-up.

## Introduction

Chronic musculoskeletal pain (CMP) affects quality of life, disability, and work [1, 2]. Workers with CMP have high rates of absenteeism and presenteeism (presenteeism: the phenomenon of people still turning up at their jobs, despite complaints and ill health that should prompt rest and absence from work [3]), with productivity losses equivalent to 1.6% of Gross Domestic Product for the Netherlands [4]. Thus, the main goal of interventions for patients with CMP and productivity loss from work is to increase work participation. Several reviews have shown that interdisciplinary vocational rehabilitation (VR) programs are effective in realizing this goal [5–7].

There is large variation in the content of VR programs [5–8]. A recent review recommended that effective VR programs should encompass the following three domains: 1. health-focused (i.e., health services intervention subcategories such as graded activity/exercise, cognitive behavioral therapy [CBT], work-hardening), 2. service coordination (i.e., improving communication within the workplace or between the workplace and the healthcare providers), and 3. work modification (i.e., modified duties, modified working hours, supernumerary replacements, ergonomic adjustments, or other worksite adjustments) (Box 1) [5]. The same review also mentioned that a multi-domain intervention including components in at least two of the three domains mentioned, can help reduce lost time from work for CMP-related conditions [5].

**Box 1:** Intervention components in rehabilitation treatments

*Health-focused interventions* These interventions facilitate the delivery of health services to the injured worker either in the workplace or in settings linked to the workplace (e.g., visits to healthcare providers initiated by the employer/workplace). Specific health services intervention subcategories for which evidence synthesis was conducted include; graded activity/ exercise, cognitive behavioural therapy, work hardening and multi-component health-focused interventions (which often included the above elements as well as: medical assessment, physical therapy, psychological therapy, occupational therapy)	
*Service coordination interventions* These interventions were designed to better coordinate the delivery of, and access to, services to assist RTW within and involving the workplace. Coordination involves attempts to improve communication within the workplace or between the workplace and the healthcare providers. Examples are development of RTW plans, case management and education and training	
*Work modification interventions* These interventions alter the organization of work or introduce modified working conditions. Examples are: workplace accommodations such as provision of modified duties, modified working hours, supernumerary replacements, ergonomic adjustments or other worksite adjustments	
*Multi-domain interventions* These interventions had multiple intervention components and included at least two of the three above intervention domains [e.g., interventions that involved graded activity in the workplace (health-focused domain) in addition to modified working conditions (work modification domain)]	

Text obtained from Cullen et al. [4]

The review mentioned above and other studies on this topic mainly consist of RCT studies in which multi-domain programs were compared with usual care [5, 6, 9] or with single component programs from the health-focused domain, such as graded activity/physical exercise [6, 8, 10], or education [6]. Other RCTs showed positive results of the additional effect on work participation of patients with acute and subacute low back pain when the work-related component was added to a multidisciplinary, health-focused program [11–14]. However, little evidence is available about the additional increase in effect on work participation when components from the work-related domains (i.e., service coordination and work modifications; Box 1) are added to a multi-component health-focused program conducted for patients with CMP. The addition of a focused work module appears not part of standard care for patients with CMP in most industrialized countries. However, the evidence concerning this niche is contradictory.

On the one hand, an RCT study conducted in Norway in patients with neck and back pain found no significant differences in work participation between the group who took part in a multidisciplinary program (i.e., multi-components from the health-focused domain) that included work-focused components and a group who only took part in a multidisciplinary program [15]. On the other hand, a retrospective cohort study conducted in Canada showed that a multidisciplinary (i.e., multi-components from the health-focused domain) pain program that included return to work coordination had 3.4 higher odds of a return to work compared with a multidisciplinary program without coordination [16].

In summary, while the evidence on the overall effectiveness of VR is robustly positive, the evidence concerning the content of VR is contradictory. In the present study, we analyzed the difference in work participation of patients who were referred to multi-component health-focused VR program with or without an additional work module in clinical practices in the Netherlands (VR+ and VR respectively).

The research question of this study was: Are patients with CMP who are on sick leave from work more likely to participate in work if they take part in a VR+ program compared with patients who only take part in a VR program? Based on recommendations from various systematic reviews to include work domains in VR to achieve successful work participation [5, 6, 9, 17], we hypothesized that patients who took part in the VR+ program would have higher odds of participating in work compared to patients who only took part in the VR program.

## Methods

The Strengthening the Reporting of Observational Studies in Epidemiology (STROBE) checklist was used in the design and reporting of this study [18].

### Design, Setting, and Procedure

A retrospective cohort study was conducted, with data collected from November 2014 to July 2018 by seven rehabilitation centers located throughout the Netherlands. These seven centers all offered interdisciplinary VR for workers with CMP who were hampered in their work participation. Patients were referred to the VR program by their occupational physician, general physician, rehabilitation physician, medical specialist, or others. Before entering the VR program, patients completed web-based questionnaires (T0) and underwent a multidisciplinary (MD) screening performed by an MD team consisting of a rehabilitation physician, psychologist, physical therapist, and vocational specialist. After the MD screening, the team and patient decided whether a VR+ program was appropriate or not (criteria, see [19]). Before VR+ started, the employer of every patient was asked to reimburse the additional work module (€1200), which was a condition of the patient participating in the VR+ program. VR was reimbursed by the healthcare insurer. Apart from the additional work module, patients of both programs participated as one group. Patients received web-based questionnaires at discharge (T1) and at 6-months follow-up (T2). If patients did not complete the T0–2 questionnaires within a week, they received a reminder by email.

### Participants

Working age individuals (18–65 years) with subacute or chronic musculoskeletal pain and reduced work participation (full or part-time sick leave) who were referred to vocational rehabilitation and who underwent a vocational rehabilitation program (VR+ or VR) between September 2014 and October 2017 participated in this study. Patients were excluded if they had no paid work, if they were not able to complete questionnaires in Dutch, or if they did not grant informed consent. The Medical Ethical Committee of the Academic Medical Center, Amsterdam, the Netherlands, authorized this study and decided that a full application was not required (Number W18_194). Participation in the study was voluntary, all participants provided informed consent, and answers were processed anonymously.

### Context

When an employee is sick-listed in the Netherlands, both the employee and employer are responsible for the work participation process during the first 2 years of sick leave. According to the Dutch Gatekeeper Improvement Act, the employer must provide wage replacement and modified work during this 2-year period [20].

### Interventions

#### Vocational Rehabilitation (VR)

The vocational rehabilitation (VR) program was an interdisciplinary group-based program that consisted of multi-components from the health-focused domain. They included general exercise therapy based on principles of graded activity (total ~ 60 h; 30 × 2 h), CBT (total ~ 7.5 h; 15 × 0.5 h), group education (total ~ 15 h; 15 × 1 h), and relaxation (total ~ 7.5 h; 15 × 0.5 h). There were two evaluation moments with the patient: one mid-evaluation after seven weeks and one end evaluation at discharge. A report from these two evaluation moments was sent to the patient. The MD team consisted of a physician, physiotherapist, and a psychologist. The program lasted fifteen weeks (total ~ 90 h) with two 3.5 to 4 h sessions per week. More information about the content of the VR program can be found in the study protocol paper [21].

#### Vocational Rehabilitation + Work Module (VR+)

The vocational rehabilitation + work module (VR+) program was an interdisciplinary group-based program that consisted of the same health-focused components as the VR program, but was extended with a work module. The work module consisted of case management and a workplace visit (total of ~ 10 h), and was executed by an RTW coordinator. The case management involved discussion of work-related problems, the design and discussion of the progress of a work participation plan, and the provision of information about work-related legislation. The company visit included communication between the patient, the RTW coordinator, and the employer with the goal of discussing and resolving barriers to and facilitators of work participation, as well as discussing a work participation plan. A workplace inspection with possible advice for ergonomic adjustment was also part of the workplace visit. There were two evaluation moments with the patient: one mid-evaluation after seven weeks and one end evaluation at discharge. A report of these two evaluation moments was sent to the patient and his/her employer and occupational physician. If necessary, the evaluation reports were discussed with the employer and/or occupational physician. The MD team consisted of a physician, physiotherapist, psychologist, and an RTW coordinator. The program lasted fifteen weeks (total ~ 100 h) with two 3.5 to 4 h sessions per week. An outline of the content and dosage of the modules of the VR+ program are described in the study protocol paper [21].

### Measures

#### Dependent Variable: Work Participation

Work participation was assessed using the *working status* item of the *imta Productivity Cost Questionnaire-Vocational Rehabilitation version* (iPCQ-VR) [22]. Working status was assessed with the question: “Are you working full-time at this moment?” with the answer categories: “Yes,” “No, I am partly at work,” and “No, I am on 100% sick leave.” In the case of patients being partly at work, there was an additional question: “How many hours are you working per week at the moment?” For the aim of this study, the *working status* and *hours working per week* items were first converted into a continuous variable of “hours working per week.” In a second step, the change in working hours per week was calculated by subtracting working hours per week at T1/T2 from the working hours per week at T0. In a final step, the working hours per week difference was dichotomized into “Achieved work participation” for those who worked at least one hour or more per week at T1/T2 compared to T0, and “Not achieved work participation” for those who worked the same working hours per week or less at T1/T2.

#### Independent Variables

The fixed independent variable in this study was *type of intervention* (VR+/VR). The other independent variables selected were potentially confounders of the outcome of “work participation.” The independent variables of this study were clustered into biopsychosocial characteristics [23]: demographic, psychological, disorder-related, and work-related. Hereafter, we briefly describe the content and score ranges of the independent variables selected and used in this study. A detailed description and clinometric properties of the questionnaires included can be found elsewhere [21, 22, 24].

### Demographic Characteristics

The following demographic characteristics were included: *age* [25–28], *gender* [16, 26–29], and *level of education* [27, 30–33]. Age was dichotomized based on the median. Level of education was divided into three categories: “low” (including primary school, lower vocational education, and lower secondary school), “medium” (including intermediate vocational education and upper secondary school), and “high” (including upper vocational education or university) [30].

### Psychological Variables

The following psychological characteristic was used: *job-related illness behavior* [30, 34, 35], was measured with a subscale from the Work Reintegration Questionnaire (WRQ) [34, 35]. The subscale consist of multiple statements which are answered on a 4-point Likert scale (1 = disagree, 2 = somewhat agree, 3 = quite agree, 4 = completely agree). The illness behavior scale ranges from 10 to 40 and was dichotomized, with scores above 34 referring to high illness behavior [34].

### Disorder-Related Characteristics

The following disorder-related characteristics were used: *duration of complaints* [16, 36], *pain intensity* [25, 27, 28, 37], *widespread pain* [26, 27, 38], *level of disability* [25, 27, 39, 40], and *perceived health* [27, 28]. Duration of complaints was dichotomized into “subacute” (duration of complaints 3 to 6 months) and “chronic” (more than six months) complaints [36]. Pain intensity was assessed on a 11-point Likert scale, as the mean pain score in the preceding week, where 0 denoted no pain and 10 denoted worst possible pain. Pain intensity was dichotomized into “high pain score” (score of ≥ 7) versus “medium/low pain score” (score of ≤ 6) [2]. Widespread pain was dichotomized into “yes” or “no.” Widespread pain was defined as “yes,” if pain in the upper extremities (arm, hand, or wrist), lower extremities (hip, knee, ankle, or foot) and axial skeletal pain (back) was present [41].

Level of disability was measured with the Pain Disability Index (PDI) [42], which is a 7-item questionnaire that measures self-reported pain-related disability. The PDI measures seven dimensions: family/home responsibilities, recreation, social activity, occupation, sexual behavior, self-care, and life support activity on a 0–10 scale (0 denotes “no disability” and 10 denotes “maximum disability”). Total scores range from 0 to 70, with higher scores reflecting higher level of disability. The level of disability score was dichotomized based on the median. Perceived health was assessed with a single health status item obtained from the RAND-36 [43, 44]: “What do you think about your health in general?,” with five answer categories, ranging from “excellent” to “bad.” Perceived health was dichotomized into good health (“excellent,” “very good,” and “good”) and moderate health (“moderate,” “bad”).

### Work-Related Characteristics

The following work-related characteristics were used: *RTW expectation* [27–30, 37, 45–47], *sick leave duration* [26, 27, 48, 49], *working status* [25, 27, 40, 50], *job strain* [32], and *job dissatisfaction* [29, 51]. RTW expectation was assessed on a 0–10 scale, with patients rating the certainty that they will be working in six months, where 0 represents “Not at all certain” to 10 “Extremely certain.” We dichotomized this item into negative RTW expectancy (score 0–5) and positive RTW expectancy (score 6–10). Sick leave duration was assessed with the sick leave long item of the iPCQ-VR questionnaire [22]. We dichotomized this item into long-term sick leave or not (“yes” = absenteeism for six weeks or more; “no” = absenteeism for less than six weeks). The decision to consider a period of 6 weeks’ sick leave in this study was based on Dutch social security legislation [52]. Working status was assessed with the working status item of the iPCQ-VR [22]. We dichotomized this item into “full sick leave” and “part-time sick leave.” Job strain and job dissatisfaction were measured with two subscales of the WRQ, which were dichotomized based on norm scores [31]. The job strain scale ranges from 7 to 28 and was dichotomized, with scores above 17 referring to high job strain. The job dissatisfaction scale ranges from 12 to 48 and was dichotomized, with scores above 30 referring to high job dissatisfaction.

### Statistical Analyses

Generalized estimating equations (GEE) analyses were applied. The analyses were performed in four steps. In the first step, a crude model was run with work participation (achieved/not achieved) as the dependent variable and type of intervention (VR/VR+) as the independent variable. In the second step, time and interaction of time and type of intervention was added to the crude model. In step three, we examined whether confounding variables were present. The model from step 2 was used, with as the reference for time: 6-months follow-up. If the regression coefficient of type of intervention increased or decreased ≥ 10%, we considered the independent variable as a confounder. Based on the available evidence, we assumed a priori that RTW expectation [27–30, 37, 45–47], work status [25, 27, 40, 50], and sick leave duration [26, 27, 48, 49] were potential confounders. In the fourth and final step, the final model was run, consisting of type of intervention, time, interaction time*intervention type and confounding variables. We report odds ratios, 95% confidence intervals of odds ratios, and p-values. All analyses were performed using SPSS Statistics for Windows, version 25.0 (2015), IBM Corp., Armonk, NY.

To visualize the relationship between type of intervention and the dependent variable work participation, working status proportions were provided for each time point (baseline, discharge, 6-months follow-up) separated for VR and VR+.

### Missing Data

The missing data mechanism (i.e., missing complete at random [MCAR] or missing at random [MAR] [53]) was analyzed by conducting a T-test and Little MCAR tests.

## Results

**Fig. 1:**
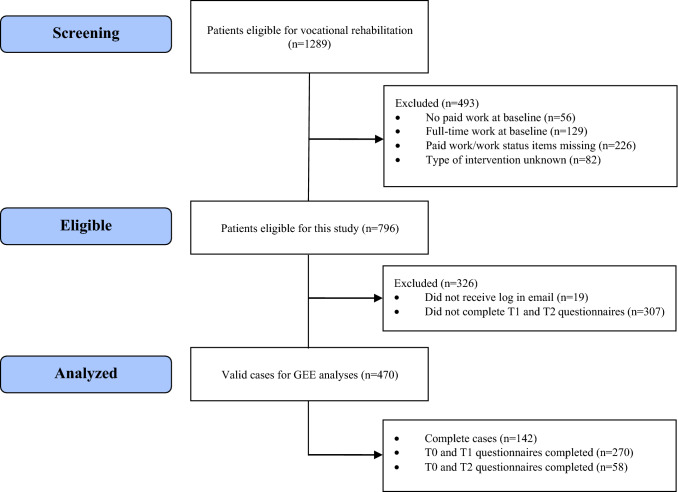
Flow chart of participants in this study. *T0* screening, *T1* discharge, *T2* 6-months follow-up, *GEE* generalized estimating equations

Out of 796 eligible patients, a total of 470 (59%) patient-data was eligible for analyses. Of these, 123 (26%) received VR and 347 (74%) VR+. Figure 1 shows a flowchart of the participant inclusion and reasons for dropout. The missing data mechanism for T1 and T2 was missing at random. The sample characteristics of both programs are presented in Table 1.

**Table 1:** Baseline characteristics of the study population (n = 470)

	VR (n = 123)	VR+ (n = 347)
	Mean (SD) or %	Mean (SD) or %
Age (years), mean	46.9 (11.3)	47.5 (10.5)
≥ 50 years (%)	49	50
Gender (% female)	53	62
Education		
Low	35	21
Medium	42	42
High	21	31
Other	3	6
Contract (h/week)	31.3 (9.8)	30.8 (9.0)
Work status		
Part-time sick leave	54	49
Full sick leave	46	51
Sick leave > 6 weeks (% yes)	45	56
Widespread pain (% yes)	21	13
Duration of complaints		
< 6 months	26	25
0.5–1 year	25	28
1–2 years	20	18
2–5 years	10	16
More than 5 years	19	14
Perceived health (% good)	55	56
Pain intensity (0–10)^a^	5.8 (2.0)	5.2 (2.4)
≥ score 7	48	39
Level of disability (PDI 0–70)^b^	36.7 (11.0)	35.6 (11.9)
≥ score 37^c^	49	49
RTW expectancy (0–10)^d^	5.5 (3.1)	6.5 (2.6)
Median	5	7
≥ score 6	49	61
Job strain (7–28)	14.9 (5.4)	15.3 (5.3)
≥ score 18	31	31
Job dissatisfaction (12–48)	24.6 (8.0)	23.1 (7.4)
≥ score 31	24	16
Job-related illness behavior (10–40)	32.7 (5.7)	31.7 (5.8)
≥ score 35	46	40

*SD* standard deviation, *PDI* pain disability index, *RTW* return to work

^a^0 = no pain, 10 = worst possible pain

^b^0 = no disability, 70 = maximum disability

^c^Median of total sample was 36

^d^0 = not at all certain, 10 = extremely certain

### Work Participation

**Fig. 2:**
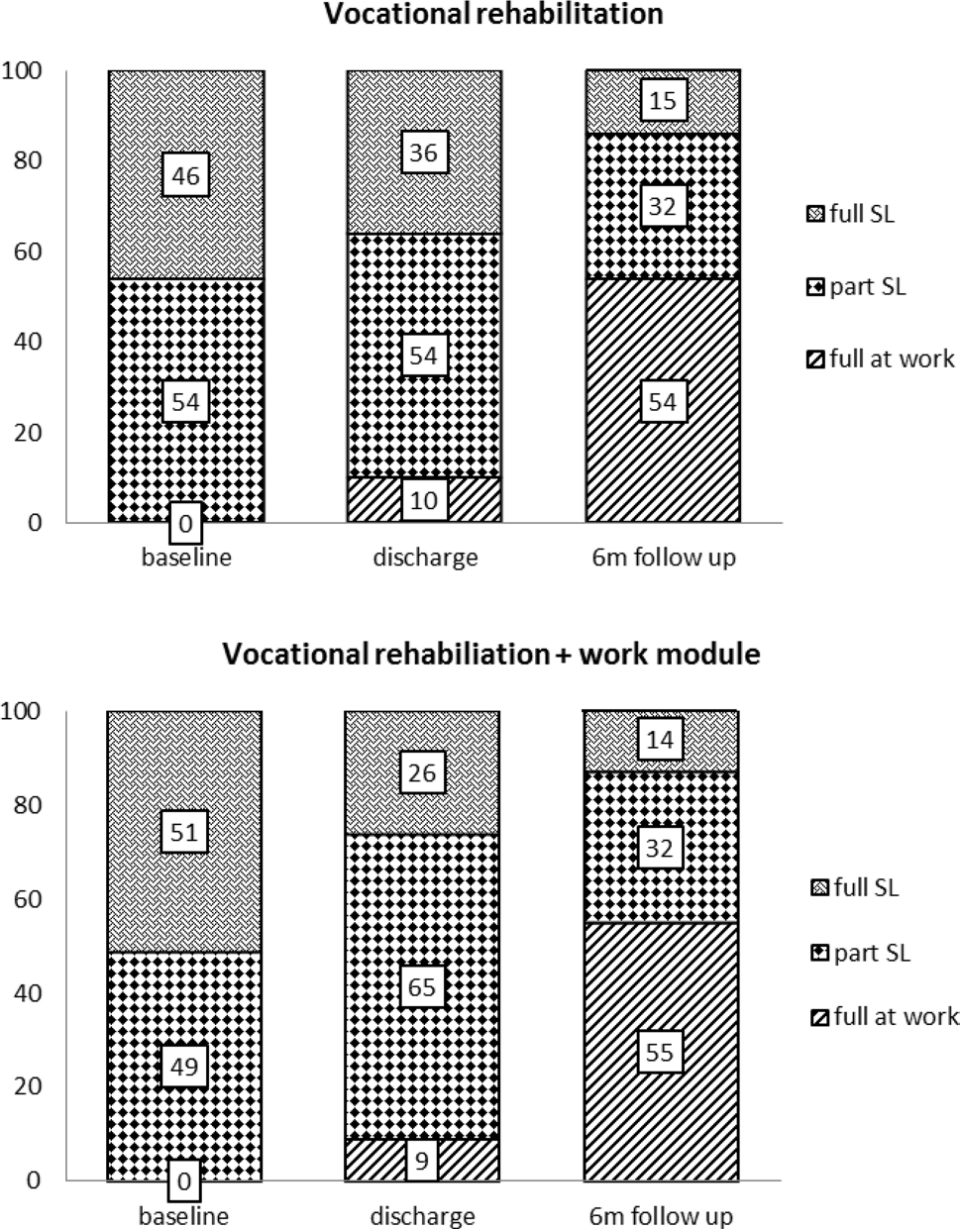
Working status proportions at baseline, discharge, and 6-months follow-up. *SL* sick leave. *Note* Due to rounding features, the total proportion per time point might deviate from 100%

At discharge from vocational rehabilitation, 64% of participants in the VR program and 74% in the VR+ program achieved work participation (Fig. 2). At 6-months follow-up, 86% of participants in the VR program and 87% in the VR+ program achieved work participation. From baseline to 6-months follow-up, work participation increased 32% in VR and 38% in VR+.

### GEE Analyses

The crude model showed a significant longitudinal relationship between VR+ on the achievement of work participation: OR 1.8 (CI 1.2–2.6), p = 0.01. The model with time and the interaction intervention*time showed a borderline non-significant relationship between VR+ on the achievement of work participation on discharge: OR 1.8 (CI 1.0–3.4), p = 0.06, and a significant relationship on 6-months follow-up: OR = 1.8 (CI 1.1–2.8), p = 0.01. RTW expectation, age, and gender showed to be confounders in step three of the analyses. The final model (Table 2), with the three confounding variables included, showed a significant relationship between VR+ on the achievement of work participation on 6-months follow-up: OR = 1.7 (CI 1.0–2.8), p = 0.04. A non-significant relationship was observed on discharge: OR = 1.3 (CI 0.6–2.5), p = 0.51. RTW expectation was the only significant confounder in the final model: OR 3.1 (CI 2.1–4.6), p = 0.00 (Table 2).

**Table 2:** Final models at discharge and 6-months follow-up

	Discharge		Six-months follow-up	
	P-value	OR (CI 95%)	P-value	OR (CI 95%)
Type of intervention	0.51	1.3 (0.6–2.5)	**0.04**	**1.7 (1.0–2.8)**^**a**^
RTW expectation	**0.00**	**3.1 (2.1–4.6)**	**0.00**	**3.1 (2.1–4.6)**
Age	0.59	0.9 (0.6–1.3)	0.59	0.9 (0.6–1.3)
Education	0.19	1.2 (0.9–1.6)	0.19	1.2 (0.9–1.6)

P-value of ≤ 0.05 in bold

^a^Original value lower bound: 1.02

## Discussion

We hypothesized that patients who received VR with an additional work module (VR+) would have greater odds of achieving work participation compared to patients who received VR without work module. Because this study demonstrated greater odds of VR+ on work participation at 6-months follow-up, our hypothesis was supported. This finding is consistent with the recommendations reported in systematic reviews to include work components to optimize work participation [5, 6, 8, 9, 17, 54, 55]. Both VR programs studied in this paper showed highly beneficial work participation rates at 6-months follow-up: 86% of the VR group were at work (full-time or part-time) and 87% of the VR+ group were at work. The work participation rates at 6-months follow-up reported in this paper are higher compared to multi-domain VR described by others, who showed mean work participation proportions of 65% ± 11% [56–62]. The high and quite similar work participation rates at 6-months follow-up might be a consequence of the Dutch social security system. Within this system, the employer has a mandatory role in offering modified work. All patients in this study had been offered this in some form, including those in the VR group. In Dutch practice, therefore, the contrast between VR and VR+ may have been smaller than in non-Dutch practice. The dosage of VR+ was similar to VR (100 and 90 h respectively), which may have also contributed to the small difference in results. The difference at discharge was non-significant, which we hypothesized was due to insufficient ‘wash-in’ of work module effects at discharge. Moreover, the results of this study may provide confirmation that work modifications can support the work reintegration of patients with CMP and sick leave from work [5]. How the three core elements (Box 1) should be delivered optimally, however, may depend on country-specific system characteristics and further study.

The present study was performed within clinical practice and studied the impact of work participation of two multi-domain programs. Other studies were conducted in a controlled setting, and frequently compared multi-domain programs with single-component programs or care as usual [5, 6, 8–10]. This complicates comparison of the findings of the present study with these studies. Nonetheless, we detected two quite similar studies compared to ours. The first study was an RCT conducted in Norway in patients with neck and back pain. This study showed deviating results compared to our study, namely no significant difference between a group who took part in a multidisciplinary program that included a work focus and a control group who only took part in a multidisciplinary program [15]. One explanation for these results might be the fact that for the multidisciplinary work-focused group it was not possible to intervene at the workplace due to regulations in Norway. The other quite similar retrospective cohort study was conducted in Canada, and showed that patients who completed a multimodal pain program that included RTW coordination had 3.4 higher odds of returning to work compared with patients who received the multimodal program without RTW coordination [16]. However, this study did not correct for RTW expectancy. Based on the present study, and many others [27–30, 37, 45–47], it is clear that RTW expectation is an important confounder in the relationship between an intervention program and a focus on improving work participation. Consequently, a clinical implication is to take RTW expectations into account at inclusion/screening before the start of an interdisciplinary VR program: patients with positive RTW expectations have higher odds of responding successfully to VR or VR+. Low RTW expectations may need further clinical attention to the underlying reason for this, and consequently, targeted intervention if modifiable.

### Strengths and Limitations

One strength of a retrospective study is its observational character, as the researcher is able to observe what actually happens or naturally occurs in practice. This is a great advantage in terms of adaptation for professionals. In addition, in our case, it was possible to correct for confounding variables which were clustered a priori based on the biopsychosocial model. This increases knowledge of which factors are important to take into account in research and clinical practice. Furthermore, because we applied GEE analyses, it was possible to include the dependency of in-person change in work participation over time in the statistical model. This increased the robustness of our findings.

One limitation of a retrospective cohort design is that the intended intervention is less controllable, which may bias the results. In our case, contamination bias between the two programs could have occurred. Patients from both intervention groups were undertaking rehabilitation together. Patients who only participated in the VR program probably obtained information from patients who completed the VR+ program and from the RTW coordinators during group meetings or coffee breaks. Because 3 out of 4 patients received the VR+ program, the chance of contamination bias, resulting in a lack of contrast, was high.

Another limitation of this study was that regarding the VR+ program it was unknown if patient actually received the components of the work module (ergonomic adjustments, case management, RTW plan) in practice. Compliance with the work module could have influenced work participation, but we were unfortunately not able to control for this factor.

Selection bias may also have occurred, as the type of program a patient participated in was dependent on the employer’s willingness to pay for the additional work module. However, at baseline there were no substantial differences between job dissatisfaction and job strain between the VR+ and VR groups. There were probably other factors which influenced the outcomes of the additional work module. From the beginning, one might expected that the VR+ group would have higher odds of achieving work participation compared to the VR group, due to differences in a number of variables: the VR group was less educated, had a higher proportion of widespread pain, higher pain scores, higher disability scores, and lower RTW expectancy. However, almost all of the independent (possible confounding) variables selected a priori were not included or did not contribute to the final models. The only significant independent variable (and also confounder) in the final models at discharge and 6-months follow-up was RTW expectation. Because a positive association of VR+ and work participation at 6-months follow-up in the final model was found, we proposed the influence of selection bias on our results was low.

One final limitation was the proportion of loss to follow-up (41%), which negatively influenced the sample size of the analyzed cases (n = 470). This might hampered the sample's representativeness with the population of interest. Post-hoc analyses with the eligible source population of n = 796 showed similar meaningful baseline characteristics compared to the analyzed cases (age, gender RTW expectancy, pain intensity, pain disability, sick leave status). Hence, we assume our findings were representative for the population of interest.

### Methodological Considerations

One methodological consideration is that we included RTW expectation as a factor in the GEE model, but we did not include “work environment” as a factor in the GEE model. It is however known that when subjects return to a workplace with a bad environment, they are more likely to fall back into sick leave again [27]. It may be hypothesized that the RTW expectation variable is not solely an individual variable, but that this variable is influenced by workplace specific variables, such as job strain and work environment. Because we did not include the latter factor in the GEE model, this might have overestimated the relationship between RTW expectation and work participation. Another methodological consideration is the high proportion of incomplete cases (i.e. only valid data on baseline-discharge or baseline-6mo follow-up; Fig. 2) which were used for the GEE analyses. However, one of the advantages of the GEE approach is that it can handle missing data very well and that data imputation techniques are not necessary to produce robust results [63].

### Clinical Implications

The results of this study suggest to add in general a work module to VR in order to optimize work participation of patients with chronic musculoskeletal pain and sick leave from work. When actions for improving the work participation are already accomplished at the workplace before entering VR, it can be considered not to add a work module to VR. This should be discussed on a patient bases at the inclusion/screening phase before VR starts.

### Future Directions

Because our study showed that patients with positive RTW expectations had three times higher odds of responding successfully after VR (independent of type of program), we recommend that future research should assess RTW expectations at baseline and correct for this variable during the analyses. Another future direction for research would be to execute return on investment analyses on the added value of work modules when nested in VR. This information is important for those who are asked to reimburse these modules.

## Conclusion

There is a positive and significant longitudinal relationship between vocational rehabilitation with additional work module, compared to VR without additional work module, on work participation of patients with chronic musculoskeletal pain and sick leave from work.
